# Tracking sources of *Clostridium botulinum* type E contamination in seal meat

**DOI:** 10.1080/22423982.2017.1380994

**Published:** 2017-10-05

**Authors:** Daniel Leclair, Jeffrey M. Farber, Franco Pagotto, Sandy Suppa, Bill Doidge, John W. Austin

**Affiliations:** ^a^ Bureau of Microbial Hazards, Health Products and Food Branch, Health Canada, Ottawa, Canada; ^b^ Nunavik Research Centre, Makivik Corporation, Kuujjuaq, Canada

**Keywords:** *Clostridium botulinum*, environment, Arctic, marine mammal, spore, meat handling

## Abstract

Botulism in Nunavik, Quebec is associated with the consumption of aged marine mammal meat and fat. The objective was to identify meat handling practices presenting a risk of contamination of seal meat with *C. botulinum*. Potential sources of contamination were assessed through interviews with *igunaq* producers from five communities of Nunavik. These sources were verified by detection and isolation of *C. botulinum* from *igunaq* prepared in the field from seal carcasses. Interviews indicated practices presenting a risk for contamination included: placing meat or fat on coastal rocks, using seawater for rinsing, and ageing meat in inverted seal skin pouches. Although the presence of *C. botulinum* type E spores was detected in only two of 32 (6.3%) meat or fat samples collected during the butchering process, two of four *igunaq* preparations from these samples contained type E botulinum toxin. Analysis of *C. botulinum* type E isolates recovered from these preparations indicated that shoreline soil may be a source of contamination. Seal meat and fat may be contaminated with *C. botulinum* type E during the butchering process. Measures can be adopted to reduce the risks of contamination in the field and possibly decrease the incidence of type E botulism in Nunavik.

## Introduction

Type E botulism is endemic in Alaska and northern Canada, where the annual incidence ranges from 6.9 to 50.5 cases per 100,000 population [1,2]. The Canadian Inuit population has been primarily exposed to botulinum neurotoxin type E via the consumption of aged marine mammal products [3,4]. Aged marine mammal meat, or *igunaq* in Inuktitut, has been implicated in several outbreaks of type E botulism, particularly in the Nunavik region of northern Quebec [5]. Walrus or seal *igunaq* is usually prepared by ageing meat and blubber (fat attached to the skin), 2–6 weeks in a traditional pouch made from the skin of the harvested animal or within a modern-type container such as a plastic bag, plastic pail, wooden box or galvanised barrel [6]. *C. botulinum* type E is common in the coastal environment of Alaska [7,8] and Ungava Bay [9], increasing the risk of contamination of marine mammal meat with type E spores during the butchering process.

The link between type E botulism outbreaks in Arctic regions and environmental sources of *C. botulinum* type E contamination has not yet been demonstrated using molecular epidemiological tools. Microbial source tracking methods such as pulsed-field gel electrophoresis (PFGE) have been previously used to track environmental sources and to study the genetic diversity of *C. botulinum* type E [9–11]. The objective of this study was to identify the sources and routes of seal meat contamination with *C. botulinum* type E spores at the butchering site. A sub-set of seal *igunaq* producers from five communities in Nunavik were interviewed to determine common meat handling practices during butchering. The safety of these practices with respect to foodborne botulism was then assessed in the field by evaluating the presence of type E spores on freshly cut meat and related *igunaq* preparations from four harvested seals and their genetic relatedness was assessed against strains from the proximate coastal environment.

## Materials and methods

### Interview with igunaq producers

The interview was designed to provide information on potential routes of contamination of seal meat with environmental and animal sources during the butchering and ageing processes of seal *igunaq*. Interviews were conducted in Inuktitut with 20 producers of seal *igunaq* from five communities in Nunavik, Quebec between 2001 and 2002. The selection of communities was based on their geographic locations and incidence of foodborne botulism [3,4,12–14]. Kuujjuaq, Kangiqsualujjuaq and Tasiujaq, three villages located along the southern shore of Ungava Bay, constituted the affected villages, since they have been the sites of several outbreaks of type E botulism. Kangiqsujuaq and Quaqtaq, two villages located on the coast of the Hudson Strait, were selected as villages with low incidence rates of botulism, as both had only one recorded botulism incident since 1967. The questionnaire was first tested in Kuujjuaq with five experienced producers of seal *igunaq*, to allow adjustments and standardization of the questions by the interviewer.

### Studies at the butchering sites

A seal hunting trip was conducted in southern Ungava Bay during the summer of 2001 to assess the potential risks of seal meat contamination with *C. botulinum* type E under field conditions. A ringed seal (*Phoca hispida*) was harvested and butchered near the mouth of the Dancelou River (N 59°06’.807’, W 68°56’.246’) situated east of the Northern village of Tasiujaq, as well as three bearded seals (*Erignathus barbatus*) in the area of Qikirtajuaq Island (N 58°17’.589’, W 67°40’.046’), at the mouth of the Whale River and east of the Northern village of Kuujjuaq. At each butchering site, the handling practices of the hunters were observed to determine if they posed a risk for contamination of meat of harvested seals with *C. botulinum* type E spores. A risk for contamination was considered significant during the butchering, if any field practices allowed the meat or fat tissues to come into contact with environmental sources of *C. botulinum* type E, such as coastal rocks, shoreline soil and seawater or intestinal materials and skin of seals. Prior to each butchering, the animal was laid on its back and the hunter proceeded to the cutting, evisceration and butchering of the carcass as normally done. Following the observation of apparent contamination resulting from a handling practice, a meat or blubber sample (average weight: 52.7 g) was collected from the affected tissues.

In addition, the coastal environment surrounding each butchering site was sampled to determine if the shoreline soil, rocks and seawater contained spores of *C. botulinum* type E at the time of butchering. Both the skin and the intestines of each harvested seal were also sampled to assess for the presence of endogenous spores, as described previously [9]. In brief, a square metre area was delineated on a large flat rock at the butchering site and swabbed using bacterial sponges. A shoreline soil sample of 300–500 grams and approximately 10 cm deep was then collected near the water line as well as a 2-litre sample of seawater within 2 feet from the shore. For each harvested seal, a 5-cm wide strip of the abdominal skin from the navel to the tail was excised prior to butchering. The entire intestinal content was gathered into the rectal segment, tied up and removed following the evisceration for testing.

### Production of conventional seal igunaq

Meat and blubber of four harvested seals were aged into *igunaq* using traditional methods, then tested for viable *C. botulinum* and botulinum toxin, to determine if the end-products were contaminated during butchering and ageing. Each carcass was cut into smaller pieces of meat and blubber and transferred into a large plastic container. These containers were either left open or covered with perforated lids. Data loggers (Optic Shuttle™, Intermountain Environmental, Inc., Logan, UT) for temperature measurements were introduced into each seal *igunaq* preparation to monitor the temperature during the ageing process. Upon arrival in Kuujjuaq, the containers were covered with an old carpet and a sheet of plywood and stored outside for the period from 19 July to 17 September. Preparations were monitored in the traditional fashion 3–4 times a week by the producer by cutting and examining a piece of aged meat for its appearance, smell and taste.

### Laboratory analysis

Detection of *C. botulinum* type E spores and/or botulinum toxin in food and environmental samples were performed by enrichment cultivation and mouse bioassay, as previously described [9]. All enrichment broths were incubated at 20°C for 7 days in an anaerobic chamber with an atmosphere of 10% H_2_, 10% CO_2_ and 80% N_2_ and then assayed for botulinum neurotoxin (BoNT) in a mouse bioassay [9]. All positive shoreline soil or sediment samples were further tested to determine the concentrations of spores using a 5-tube most probable number (MPN) procedure [9] combined with a slot blot immunoassay for type E botulinum toxin [15]. Three 10-fold-decreasing amounts (30 g, 3 g and 0.3 g) and five culture tubes for each amount, for a total of 15 tubes per environmental sample, were cultured in tryptone peptone glucose yeast extract broth. Isolation of *C. botulinum* type E from positive cultures was conducted on selective *Clostridium botulinum* Isolation (CBI) agar plates [16] using a colony blot immunoassay [17]. Up to three confirmed colonies were purified and kept for PFGE analysis using SmaI and XhoI [18].

## Results

### Interviews of igunaq producers

Twenty producers of seal *igunaq*, from five villages of Nunavik, were interviewed. Nineteen producers were men, with an average age of 62 years (range = 41–84 years) and one was a 66-year old woman. Most producers (16 of 20) had more than 30 years of experience in the production of seal *igunaq*. The questions were not consistently answered by all producers and, hence, the denominator varies among questions. Most (14 of 20) *igunaq* producers used specific shoreline sites for post-harvest meat handling activities (Table 1). Large and flat coastal rocks were preferred, but some hunters from Ungava Bay also used pebble beaches as butchering sites.

**Table 1.** Frequency of selected handling practices used by local *igunaq* producers.

		Frequency in selected Northern Villages		
Sources	Handling practices	Ungava Bay (n=3)	Hudson Strait (n=2)	All (n=5)
Coastal rocks	Repeated use of specific butchering sites	75% (9/12)	63% (5/8)	70% (14/20)
	Cutting seal on coastal flat rocks	100% (12/12)	100% (7/7)	100% (19/19)
	Placing meat and blubber on coastal rock surfaces	100% (12/12)	100% (8/8)	100% (20/20)
Shoreline soil	Cutting seal on a pebble beach	33% (4/12)	0% (0/7)	21% (4/19)
Seawater	Rinsing carcasses, meat and blubber with seawater	100% (12/12)	100% (8/8)	100% (20/20)
	Rinsing meat carriers with seawater	100% (12/12)	100% (7/7)	100% (19/19)
	Rinsing the skin with seawater	100% (12/12)	100% (7/7)	100% (19/19)
Intestinal contents	Leakage of faecal material during evisceration	17% (2/12)	0% (0/8)	10% (2/20)^a^
	Rinsing hands after emptying the intestines	100% (12/12)	100% (8/8)	100% (20/20)
	Transport of intestines with meat in the same container	50% (6/12)	0% (0/7)	32% (6/19)
Skin	Transport of flippers with meat in the same container	100% (12/12)	71% (5/7)	90% (17/19)
	Use of skin pouch (*puurtaq*) as a container for ageing	67% (8/12)	25% (2/8)	50% (10/20)

^a^Two *igunaq* producers stated that accidental leakage of faecal material occurs occasionally.

Following bleeding the seal, hunters began pelting by cutting along the median line and detaching the skin from the carcass, leaving the pelt on the rock to prevent direct contact of the carcass with the rock surface. The hunters opened the thoracic and abdominal cavities, trying not to perforate internal organs, then proceeded with evisceration. Only two of the 20 producers stated that the contents of the intestinal tract may accidentally contaminate the carcass following detachment of the rectum from the anus. These producers use seawater to rinse the faecal material from the carcass. Following evisceration, seal carcasses are cut into large pieces and placed on coastal rocks. All interviewed producers reported placing meat and fat on coastal rock surfaces and rinsing meat, fat and pelts with seawater. The meat and fat are usually transported in plastic containers by all interviewed producers, but 32% of them, all from Ungava Bay, could carry the emptied intestines in the same container.

Fifty per cent (10/20) of the *igunaq* producers occasionally used a skin pouch as a natural container for ageing seal meat and blubber into *igunaq*, but the majority (18/20) used plastic containers, plastic bags or wooden boxes. When asked if intestines or other internal organs were included with meat and fat, none of the 16 respondents included intestines or other internal organs with meat and fat, but two of the 16 respondents included flippers. In the traditional preparations, the skin of the pouches is inverted, placing fur in contact with meat or fat. The storage area or covers used to protect the preparations from the environment are diverse and include pebble, rocks, wooden boxes, plywood, fabric, sand, under a house or buried in a pebble beach. Eleven of the 15 respondents indicated soil was occasionally observed contaminating igunaq preparations.

### Safety of seal igunaq

All four igunaq preparations were stored at the same location in Kuujjuaq. Igunaq No. 1 was prepared from seal No 1, igunaq No 2 from seal No 2, etc. The average temperature of the *igunaq* preparations was 10°C and ranged from 2–23°C during the 2-month period. Based on appearance and smell, the producer assessed that the *igunaq* preparations derived from seal Nos 1 and 2 were unfit for consumption. However, the analysis of the *igunaq* for type E botulinum toxin revealed the preparations made with meat and fat from seal Nos. 1 and 2 were negative for botulinum toxin, while those made from seal Nos. 3 and 4 were positive (Table 2). Type E botulinum neurotoxin was detected in one meat extract and two oil extracts (or *misiraq*) of *igunaq* No. 3, respectively, while all 13 extracts of meat and oil samples from *igunaq* No. 4 contained type E botulinum toxin (Table 2).

**Table 2.** Sources and contamination of seal meat with *C. botulinum* type E at the butchering site and toxicity of related *igunaq*.

		Environmental sources of *C. botulinum*			Contaminated raw tissues		Toxicity of *igunaq*^c^	
Seal ID	Butchering site	Shoreline soil	Seawater	Coastal rock	Meat	Fat	Meat	Oil
No. 1	Flat rock at high tide	+^a^	−	−	0/1^b^	1/1	0/10	0/3
No. 2	Flat rock at high tide	+	−	−	0/4	0/5	0/10	0/3
No. 3	Flat rock at high tide	+	−	−	0/7	0/6	1/10	2/3
No. 4	Mud bed at low tide	+	+	−	0/4	1/4	10/10	3/3

^a^± Positive/negative for *C. botulinum* type E spores.

^b^Number of positive samples/total number of samples.

^c^An *igunaq* preparation is typically made of meat and fat which dissolves in oil during the ageing process. Toxicity of *igunaq* was assessed for type E botulinum toxin.

### Source tracking C. botulinum type E contamination of igunaq

Seal No. 1

**Figure 1.**
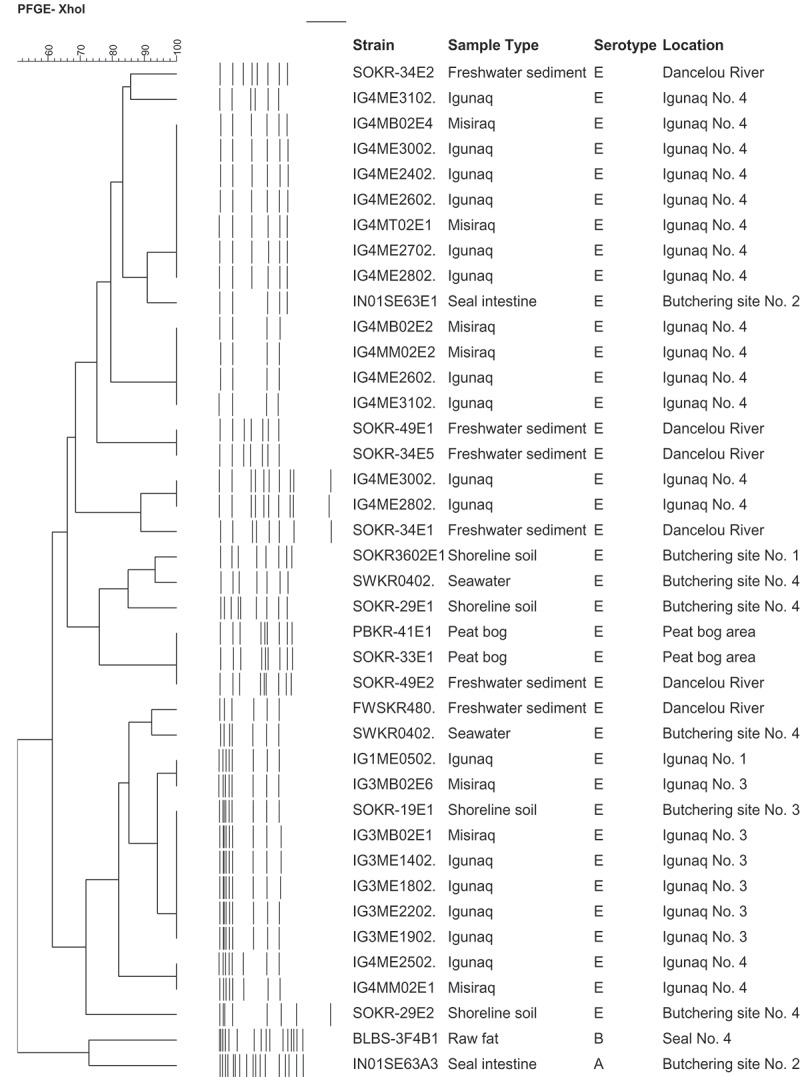
Dendrogram of PFGE analysis showing the relationship between *C. botulinum* type E isolates recovered from harvested seals and related *igunaq* preparations and those from the proximate coastal environments. SmaI was also used on all strains (data not shown). SmaI analyses generated 14 different PFGE profiles, whereas XhoI generated 20 profiles and was deemed more discriminatory. The sample types *igunaq* and misiraq correspond to the aged meat and oil components of the *igunaq* preparation, respectively. The similarity (%) among strains was determined using the Dice Coefficient and the clustering was performed by unweighted pair group method with arithmetic mean (UPGMA).

The first harvested seal (seal No. 1) was a ringed seal which was butchered on a large flat rock at high tide near a campsite near the mouth of the Dancelou River. Seal No. 1 was pulled out of the water, placed in a large plastic container and carried onto a large flat rock. Following the evisceration, the carcass was cut in large pieces of meat and fat tissues by the hunter and placed onto the surface of the coastal rocks aside from the animal prior to be transferred to a plastic container. Spores of *C. botulinum* type E were found in the surrounding shoreline soil at a concentration of 15 spores/kg, but not in the seawater or on the surface of the coastal rock where the carcass was butchered (Table 2). The origin of coastal spores was investigated by sampling two aquatic systems surrounding the campsite, a peat bog area and the Dancelou River. Two of eight sediment samples from the peat bog area and all three sediments from the Dancelou River contained type E spores at concentrations ranging from 5–89 spores/kg. None of the nine strains of *C. botulinum* type E recovered from the peat bog area or the Dancelou River shared PFGE profiles with the single shoreline soil isolate (SOKR3602E1) where seal No. 1 was butchered. Of these nine type E isolates, eight distinguishable PFGE types were identified with both SmaI and XhoI restriction enzymes, indicating a high degree of genetic diversity (Figure 1). Two clonal type E isolates in the peat bog situated closer to the river differed from a river strain by two bands with SmaI (>88% similarity level).

Of the two raw meat and raw fat samples collected during butchering of seal No. 1, one fat sample tested positive for *C. botulinum* type E; however, the preparation was not toxic (Table 2).

None of the environmental strains were closely related to the isolates recovered from *igunaq* No 1. In fact, the PFGE patterns of six isolates recovered from *igunaq* No. 1 were all indistinguishable from the PFGE pattern of a type E strain isolated from *igunaq* No. 3. These results suggest possible cross-contamination of the type E isolate from *igunaq* No. 3 to *igunaq* No. 1 at a late stage of the ageing process in Kuujjuaq, since none of the samples from *igunaq* No. 1 contained type E botulinum toxin.

Seal Nos. 2 and 3

Seal numbers 2 and 3 were bearded seals and were hunted at the mouth of the Whale River. Both were directly pulled out of the water from the shore and butchered on large flat rocks on the shore of Qikirtajuaq Island at high tide. Following the evisceration, the carcasses were cut in large pieces of meat and fat tissues placed onto the surface of the coastal rocks aside from the animals, resulting in the contamination of the surface of several pieces of tissues with sediment particles. In addition, some of the meat from seal No. 2 was found to be in direct contact with tidal water present in depressions of coastal rocks. Seawater enclosed in depressions of coastal rocks following the receding tide may contain spores of *C. botulinum* type E. Such water was used by the hunter to wash off the blood covering the meat of seal No. 3 after the butchering process. Following rinsing, all the large pieces of meat were hooked and dragged across the rocks and then piled up on the surface of coastal rocks away from the water line. This handling practice also favours contact between residual spores on the surface of the rocks and the meat. *C. botulinum* type E was found in the shoreline soil (accessed at low tide) at a concentration of 130 spores/kg, but not in the seawater or on the coastal rocks where the seals were butchered.

Of 22 meat and fat samples collected from seal Nos. 2 and 3 during butchering, *C. botulinum* type E spores were not detected, despite the tissues being placed on rocks and rinsed with seawater (Table 2).

The igunaq prepared from seal No. 2 was not toxic and *C. botulinum* type E was not recovered from the igunaq (Table 2). Unlike the igunaq prepared from the second seal, three oil samples and six meat pieces of *igunaq* No. 3 tested positive for *C. botulinum* type E. PFGE analysis of these isolates showed that the igunaq from seal No. 3 was predominantly contaminated with a single genotype (Figure 1). Cluster analysis of PFGE patterns generated from *igunaq* No. 3 isolates and those from the Whale River environment showed that the predominant genotype from *igunaq* No. 3 was also present in the shoreline soil (SOKR-19E1) collected near the butchering site of seal No. 3. These data suggested that the source of contamination of *igunaq* No. 3 was most likely of environmental origin.

Seal No. 4

Seal number 4, also a bearded seal, was butchered near the same location on Qikirtajuaq Island, but at low tide on a mud bed containing *C. botulinum* type E at 1,800 spores/kg and where the seawater from the rising tide was also positive for type E spores.

All the meat and fat tissues cut from seal No. 4 were directly transferred to the plastic container. All eight meat and fat samples collected from the bulk container following butchering were negative for type E spores, but one fat sample harboured a group I type B strain of *C. botulinum*.

Both the *igunaq* No. 4 and the related shoreline environment contained multiple unrelated type E strains, corroborating the existence of a heterogeneous population of *C. botulinum* type E in the coastal environment. Based on XhoI macrorestriction patterns, five and four distinguishable genotypes were observed in *igunaq* No. 4 and environmental samples, respectively (Figure 1).

### Skin and intestinal contents of seals

Of the three abdominal skin samples collected along the median line where the animals were incised, none harboured *C. botulinum* type E. The intestinal tract of each seal was removed intact, and none were observed to be leaking inside the carcass during evisceration. The intestinal contents of seal No. 2 contained spores of *C. botulinum* type A and type E, while the samples removed from the intestines of the other seals tested negative for *C. botulinum*.

## Discussion

Most hunters preferably select a large and flat rock in the coastal environment to butcher seals. The risk of contamination of seal meat from environmental sources is likely less when the animal is butchered on a flat coastal rock than on a pebble beach or a shoreline area covered by mud at low tide. The first meat handling practice observed in the field that may pose a risk for contamination consisted of placing meat and fat used for the preparation of *igunaq* onto the surface of coastal rocks while performing the butchering. Although the coastal rocks appeared visually clean, the surface may harbour viable spores left by the tide water [9]. The practice of placing meat and fat on coastal rocks may have led to the contamination of seal No. 1 during butchering. This practice is used by all interviewed producers of *igunaq*. The use of a clean tarp as a physical barrier under the seal, and a plastic container to place the meat and fat tissues following cutting, may reduce the likelihood of contamination with bacterial spores from shoreline soil and rocks. Up to 13 out 16 (or 81%) interviewed producers reported that they use tarp to prevent contamination from shoreline soil, but the frequency of use was not assessed.

The practice of butchering seals in the mud is probably not common among hunters, but it should be discouraged due to the high risk of meat contamination. This was confirmed by the isolation of up to seven different genotypes of *C. botulinum* type E in *igunaq* No. 4. None of the igunaq isolates shared PFGE profiles with isolates from seawater and shoreline soil samples collected near the butchering site, suggesting the existence of a high genetic diversity of *C. botulinum* type E in the environment of Whale River. The highest concentrations of spores in the coastal environment were previously found near butchering sites located along the mouths of large rivers [9]. The simultaneous contamination of foods by more than one genotype of *C. botulinum* type E has been reported in fresh fish and fishery products from Finland [19].

Despite the existence of a high biodiversity of *C. botulinum* type E in the environment of Whale River, a shoreline soil isolate of *C. botulinum* type E recovered near the butchering site of seal No. 3 was found to be indistinguishable from multiple type E isolates recovered from *igunaq* No. 3 when using cluster analysis of PFGE patterns. In contrast to *igunaq* No. 4, *igunaq* No. 3 was primarily contaminated with a single strain of *C. botulinum* type E. As seal No. 3 was butchered on a flat coastal rock at high tide, contamination of meat could have occurred either from contact with residual spores present on the rock left by the previous tide, or from spores in water that were carried onto the rock while pulling the animal out of the water. Contamination of seal meat from seawater containing spores of *C. botulinum* type E was previously suspected when spores were detected in coastline water from western Alaska near areas where marine mammals are usually butchered [8]. Rinsing seal meat or immersing the entire carcass in seawater to remove blood or to provide meat with a salty taste could also lead to the contamination of meat with spores present in seawater. This practice was used by all interviewed *igunaq* producers and was observed once in the field at Whale River where meat from seal No. 3 was rinsed with seawater taken from the depression of a coastal rock. In this case, spores of *C. botulinum* type E were not detected in the rinse water.

According to the interviews of the producers of *igunaq*, accidental contamination of seal meat with intestinal material does not occur frequently during evisceration. No evidence of leakage of the intestinal materials, into the carcass or beside the four butchered seals, was observed in the field. Hand rinsing may reduce the probability of cross-contamination of meat from intestinal contents after manually emptying the intestines before cutting the meat and fat. The intestines of the four seals were transported separately from the meat during the field observation, but 50% of interviewed *igunaq* producers from Ungava Bay stated that they occasionally transport the intestines and the meat in the same container. Apart from this handling practice, there were no regional differences showing a higher or lower risk for contamination from environmental or endogenous sources of spores during the butchering process. Although the prevalence of *C. botulinum* spores in the intestinal tract of seals and other marine mammals is low [8,9], on certain occasions these meat handling practices may lead to cross-contamination events.

A different genotype of *C. botulinum* type A was previously isolated from the shoreline soil sample collected near the Northern village of Kuujjuaraapik [9]. This was the first report of *C. botulinum* type A being detected in the Canadian sea coasts. No outbreaks of botulism have been caused by *C. botulinum* types A or B in Nunavik. In Alaska, types A and B are considered indigenous and have been reported as aetiologic agents of botulism [20,21].

The *igunaq* preparations were aged in pails and kept outdoors in Kuujjuaq from mid-July to mid-September. The average temperature of the preparations was well above the minimum growth temperature of *C. botulinum* type E (3°C) during the ageing process. Once the natural incubation was complete, the four preparations were also tested for redox potential, pH and water activity, all of which were adequate to support the growth of *C. botulinum* type E (data not shown). Botulinum toxins were detected in two preparations which were originally considered edible by the producer, while the two non-toxic preparations were perceived as unfit for food consumption. Producers commonly monitor their preparations several times a week to determine if the *igunaq* is ready to eat, either by smelling or tasting the meat or assessing if the fat is dissolved or the hairs from the skin are peeled off. However, this study shows that a producer cannot rely solely on organoleptic evaluation to assess the safety of *igunaq* preparations. The use of plastic bags or sealed containers in the ageing process of fish and marine mammals was recognised as a hazardous practice in Alaska and was discouraged [22]. Traditional methods that promote slow ageing of native foods at low temperature are encouraged over methods using modern-type containers placed above ground or inside a shed, which enables foods to age more rapidly at higher temperatures [21].

## Recommendations

To prevent contamination of seal carcasses, meat and fat with *C. botulinum* type E during butchering, a number of simple measures can be applied. Marine mammals should be butchered on rock surfaces and mud or soil should be avoided. The marine mammal should be placed on a physical barrier such as a tarp and all meat and fat should be placed in clean plastic containers immediately after cutting. Rinsing meat or any parts of the carcass with shoreline water after butchering should be avoided and the seal intestines and flippers should be transported separately from meat and fat as they could be potential sources of botulinum spores.
